# Upregulation of VSIG4 in Type 2 Diabetic Kidney Disease

**DOI:** 10.3390/life12071031

**Published:** 2022-07-11

**Authors:** Sang Youb Han, Jung Yeon Ghee, Jin Joo Cha, Young Sun Kang, Dae Young Hur, Han Seong Kim, Dae Ryong Cha

**Affiliations:** 1 Department of Internal Medicine, Inje University, Ilsan-Paik Hospital, Goyang 10380, Korea; 2 Department of Internal Medicine, Korea University, Ansan Hospital, Ansan 15355, Korea; gheejy@nate.com (J.Y.G.); minipearl@korea.ac.kr (J.J.C.); starch70@korea.ac.kr (Y.S.K.); 3 Department of Anatomy and Tumor Immunology, Inje University College of Medicine, Busan 47392, Korea; anatohur@gmail.com; 4 Department of Pathology, Inje University, Ilsan-Paik Hospital, Goyang 10380, Korea; hskim@paik.ac.kr

**Keywords:** diabetes, kidney, VSIG4, fibrosis

## Abstract

Fibrosis is the final common finding in patients with advanced diabetic kidney disease. V-set Ig domain containing 4 (VSIG4) is related to fibrosis in several diseases. It also contributes to fibrosis under high-glucose conditions in renal tubule cells. To determine the role of VSIG4 in type 2 diabetes, we examined VSIG4 expression in a type 2 diabetic animal model and podocyte. Urinary excretion of albumin and VSIG4 was significantly higher in db/db mice than in the control group. Urine VSIGs levels for 6 h were about three-fold higher in db/db mice than in db/m mice at 20 weeks of age: 55.2 ± 37.8 vs. 153.1 ± 74.3 ng, *p* = 0.04. Furthermore, urinary VSIG4 levels were significantly correlated with urinary albumin levels (r = 0.77, *p* < 0.01). Intrarenal VSIG4 mRNA expression was significantly higher in db/db mice than in control mice (1.00 ± 0.35 vs. 1.69 ± 0.77, *p* = 0.04). Further, VSIG4 expression was almost twice as high in db/db mice at 20 weeks of age. Intrarenal VSIG immunoreactivity in db/db mice was also significantly higher than that in control mice. In cultured podocytes, both high glucose and angiotensin II significantly upregulated the expression of VSIG4 mRNA and protein. In conclusion, VSIG4 was upregulated in an animal model of type 2 diabetes and was related to albuminuria and pro-fibrotic markers. Considering these relationships, VSIG4 may be an important mediator of diabetic nephropathy progression.

## 1. Introduction

Diabetic kidney disease (DKD) is one of the main microvascular complications and is the most common cause of end-stage kidney diseases worldwide. Several mechanisms have been suggested for DKD pathogenesis including advanced glycation end products, reactive oxygen species, hypoxia, and inflammation [1]. These pathologic changes ultimately contribute to renal fibrosis. Fibrosis is the final common finding in patients with advanced DKD [2]. In particular, epithelial–mesenchymal transition (EMT) has been suggested as one of the fibrotic pathways in DKD progression [3].

V-set Ig domain containing 4 (VSIG4), a member of the B7 superfamily, is a complement receptor for complement 3 and is mainly expressed in macrophages [4]. VSIG4 inhibits T cell proliferation and regulates T cell survival [5]. Recent data have demonstrated the role of VSIG4 in EMT and fibrosis in several diseases. VSIG4 is highly expressed in several malignant tumors such as lung cancer and promotes EMT in glioblastoma [6,7,8]. Further, VSIG4 accumulation correlates with aging [9].

Limited studies have shown the role of VSIG4 in kidney diseases. VSIG4 inhibits intrarenal T cell infiltration and attenuates kidney injury in a unilateral ureteral obstruction model [10]. In contrast, latent membrane protein 1 and high glucose upregulate VSIG, inducing EMT in human renal tubular epithelial cells [11,12]. Furthermore, VSIG4 is a downstream mediator of transforming growth factor-β (TGF-β) in high glucose-induced renal tubular fibrosis. Therefore, the present study was designed to determine the role of VSIG4 in a well-characterized animal model of type 2 diabetes [13].

## 2. Methods

### 2.1. Animal Studies

Eight-week old male C57BL/6J db/db mice (n = 6) and non-diabetic db/m mice (n = 6) (Jackson Laboratory, Sacramento, CA, USA) were used in this experiment. The mice were maintained on freely provided standard food and tap water and housed in a temperature- (23 ± 2 °C) and humidity-controlled (55 ± 5%) room under a 12 h dark/light cycle. All mice were placed in an individual metabolic cage before euthanasia to collect urine for 6 h. Mice were anesthetized by intraperitoneal injection of tribromoethanol (Avertin 50 mg/kg) after 12 weeks. Kidneys were weighed and stored at −80 °C until further use. Animal care and experiments were carried out in accordance with the National Institutes of Health guidelines and were approved by the Korea University Institutional Animal Care and Use Committee (KOREA-2020-0036).

Plasma levels of glucose and creatinine were measured using a glucose oxidase-based method and a modified Jaffe method, respectively. HbA1c levels were determined using an IN2IT analyzer (Bio-Rad Laboratories, Hercules, CA, USA). Urinary levels of albumin (ALPCO, Westlake, OH, USA) as well as urinary and serum levels of VSIG4 (Bioassay Technology Laboratory, Shanghai, China) were determined by enzyme-linked immunosorbent assay using commercial kits according to the manufacturer’s instructions.

### 2.2. Podocyte Culture

Mouse podocytes were used to evaluate the effect of high glucose and angiotensin II on VSIG4 expression. Cell cultures were performed as described previously [14]. Briefly, subconfluent podocytes were exposed to serum-starvation (1.0%) conditions for 24 h, and the media were replaced with fresh medium. The cells were then treated with 30 mM D-glucose or 100 nM angiotensin II for 12 and 24 h. All experiments were performed using three technical replicates.

### 2.3. Semiquantitative Reverse Transcriptase–Polymerase Chain Reactions

Total RNA extraction from the kidney tissues and quantitative RT-PCR were performed as described elsewhere [14]. Briefly, total RNA was isolated using TRIzol, and cDNA was prepared using SuperScript™ II Reverse Transcriptase (Invitrogen Carlsbad, CA, USA). Quantitative RT-PCR was performed on a LightCyler 1.5 system (Roche Diagnostics Co., Indianapolis, IN, USA) using SYBR Green with the following conditions: 10 min at 50 °C and 5 min at 95 °C, followed by 22–30 cycles of denaturation for 10 s at 95 °C and annealing with extension for 30 s at 60 °C. The expression of each gene was normalized to the mRNA expression of β-actin. The mRNA primer sequences are shown in Supplementary Table S1.

### 2.4. Immunohistochemical Staining for VSIG4, TGβ1, PAI-1, and Type IV Collagen

Paraffin-embedded kidney sections (4 µm thick) were stained with hematoxylin and eosin (H&E). Mesangial expansion and glomerulosclerosis were determined semi-quantitatively using PAS-stained sections according to a previously described method [15]. To determine the degree of mesangial matrix expansion, mesangial matrix area per glomerulus was quantified semi-quantitatively as follows: 0, 0%; 1, 0–25%; 2, 25–50%; 3, 50–75%; and 4, >75% [14].

The kidney sections to be used for immunostaining were transferred to a 10 mmol/L citrate buffer solution at pH 6.0. The sections were subjected to antigen retrieval as follows: VSIG4 and TGF-β1, 80 °C for 30 min; for type IV collagen, 1 M EDTA buffer solution (pH 8.0); plasminogen activator inhibitor-1 (PAI-1), Biogenex Retrievit (pH 8.0) (InnoGenex, San Ramon, CA, USA) and microwaved for 20 min. Endogenous peroxide activity was blocked using 3.0% H_2_O_2_ in methanol for 20 mi., and the slides were incubated at room temperature for 20 min with normal goat serum (VSIG4, TGF-β1, and type IV collagen) or 10% PowerBlock (PAI-1). Next, the following primary antibodies were incubated: mouse polyclonal VSIG4 (1:200, R&D system, Minneapolis, MS, USA) 4 °C overnight; rabbit polyclonal anti-TGF-β1 antibody (1:200; Santa Cruz Biotechnology) for 2 h; rabbit polyclonal anti-type IV collagen (1:200; Santa Cruz Biotechnology, Santa Cruz, CA, USA) for 1 h; rabbit polyclonal anti-PAI-1 antibody (1:50; American Diagnostica, Stamford, CT, USA) 4 °C overnight. The sections were then treated using an anti-goat HRP-DAB cell and tissue staining kit (R&D system) and counterstained with Mayer’s hematoxylin.

### 2.5. Protein Extraction and Western Blot Analysis

Protein was extracted from the renal cortical tissue and podocytes using NE-PER Nuclear and Cytoplasmic Extraction reagents (Thermo Scientific, Rockford, IL, USA). Then, 10 µg of protein was separated on a 4–15% SDS-PAGE gradient gel. After transfer to the polyvinylidene difluoride membrane, they were incubated with rabbit polyclonal VSIG4 (1:500, Abcam Inc, Cambridge, MA, USA) and mouse monoclonal anti-β actin (1:10,000, Sigma-Aldrich, St. Louis, MO, USA) at 4 °C overnight. After incubation with an HRP-labeled secondary antibody (1:1000), the signals were visualized using enhanced chemiluminescence reagents (Amersham, Buckinghamshire, UK).

### 2.6. Statistical Analysis

All data were analyzed using the statistical program SPSS version 25 (IBM Corp, New York, NY, USA). Data were expressed as mean ± SD. Statistical difference was analyzed using Student’s *t*-test. The correlation between urinary VSIG4 levels and urinary albumin levels was analyzed using Pearson’s correlation test. P values less than 0.05 were considered statistically significant.

## 3. Results

### 3.1. Biochemical Parameters in Experimental Animals

As expected, there were significant differences in the baseline characteristics between db/db and db/m mice at 8 weeks of age (Table 1). The db/db mice were significantly heavier in body weight and had higher fasting glucose levels. These differences were more prominent at 12 and 20 weeks. HbA1c levels showed a similar pattern at 12 and 20 weeks.

### 3.2. Renal Function and Albuminuria

There was no significant difference in the baseline urinary albumin excretion between the two groups at 8 weeks. Urinary albumin excretion for 6 h was significantly higher in the diabetic mice than in the control group starting from 12 to 20 weeks of age (Table 1). Serum creatinine concentrations were significantly higher in db/db mice than in the control mice at 20 weeks of age (0.58 ± 0.08 vs. 0.48 ± 0.06, *p* = 0.03).

### 3.3. Urinary Excretion of VSIG4

**Figure 1:**
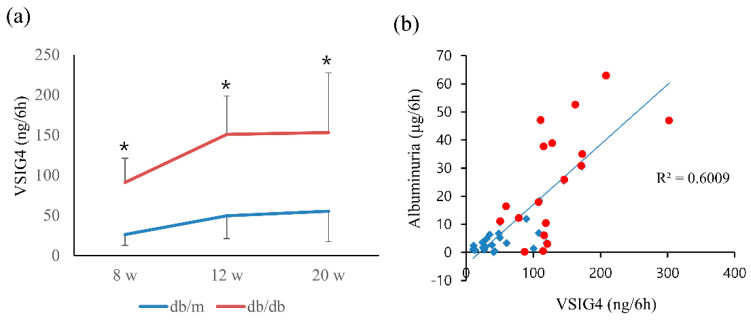
Urinary VSIG4 level and its correlation with albuminuria in db/m (n = 6) and db/db (n = 6) mice. (**a**) Urinary VSIG4 level for 6 h at 8, 12, and 20 weeks of age. (**b**) Correlation between urinary VSIG4 levels and albuminuria. Blue: db/m mice, red: db/db mice. * *p* < 0.05 vs. db/m.

Urinary VSIG4 levels were significantly higher in db/db mice than in db/m mice at 12 and 20 weeks (Figure 1a). Urinary VSIG4 levels for 6 h were approximately three-fold higher in db/db mice than in db/m mice at 20 weeks of age (55.2 ± 37.8 vs. 153.1 ± 74.3 ng, *p* = 0.04). The VSIG4 level was significantly different at the baseline even though the urinary albumin level showed no difference. Urinary VSIG4 levels were significantly correlated with the urinary albumin levels when all the levels were combined (Figure 1b).

### 3.4. Serum Levels of VSIG4 in Diabetic Mice

**Figure 2:**
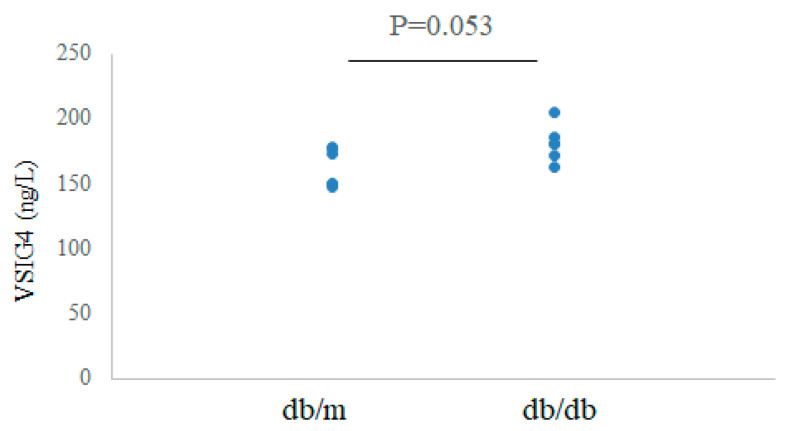
Serum VSIG4 levels in db/m (n = 6) and db/db (n = 6) mice.

The serum levels of VSIG4 protein were measured by ELISA. The levels tended to be higher in db/db mice than in control mice at 20 weeks: 181.4 ± 14.5 vs. 162.9 ± 14.8 ng/L, *p* = 0.053 (Figure 2).

### 3.5. Expression of Intrarenal VSIG4 in Diabetic Mice

**Figure 3:**
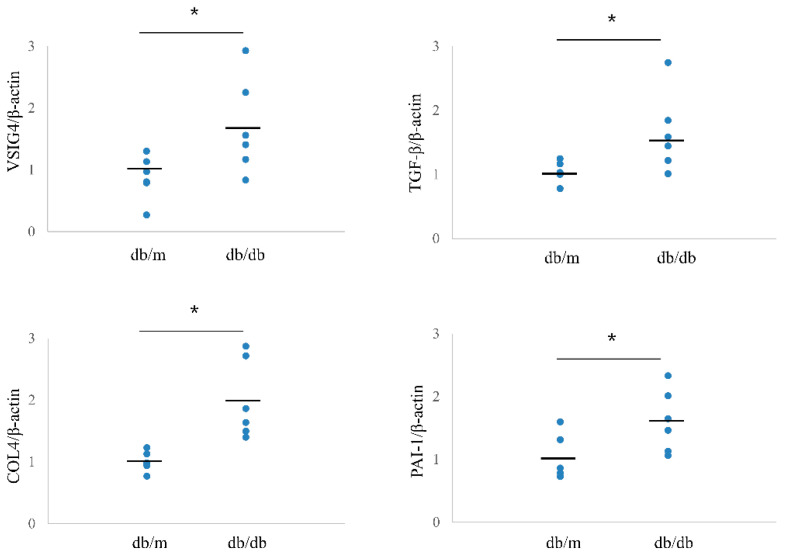
Intrarenal mRNA expression of VSIG4 and profibrotic markers in db/m (n = 6) and db/db (n = 6) mice. Black bars indicate mean value. * *p* < 0.05 vs. db/m mice.

**Figure 4:**
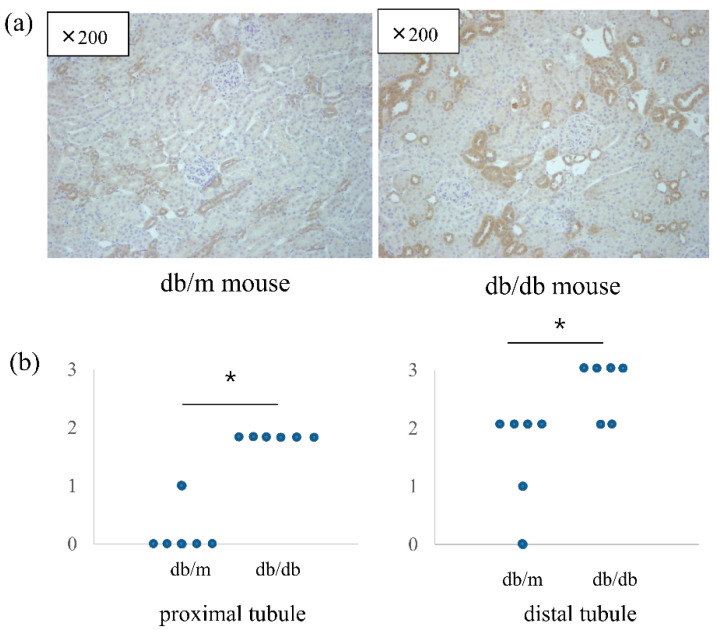
Immunohistochemical staining of VSIG4 in db/m (n = 6) and db/db (n = 6) mice. (**a**) Representative histologic findings of intrarenal VSIG4 protein expression. (**b**) Semiquantitative expression of VSIG4 in proximal and distal tubules. * *p* < 0.05 vs. db/m mice.

The intrarenal mRNA expression of VSIG4 was significantly upregulated in db/db mice compared to that in control mice. In accordance with urinary VSIG4 excretion, VSIG4 gene expression was almost two-fold higher in db/db mice at 20 weeks of age (Figure 3). Intrarenal VSIG immunoreactivity was similar to that the mRNA expression trend. Immunoreactivity of VSIG4 was clearly increased in the diabetic kidney (Figure 4). Further, immunoreactivity was prominent in the distal tubular epithelium. In the proximal tubule, the expression was weaker but was still significantly different between the two groups. However, this was not accompanied by an increase in the glomerular VSIG4 level. In addition to immunostaining, VSIG4 protein expression was validated by Western blot analysis (Supplementary Figure S1). The results were similar to the findings of immunostaining.

### 3.6. Profibrotic Markers

Intrarenal mRNA expression of profibrotic markers showed a pattern similar to that of VSIG4. The mRNA levels of TGF-β, PAI-1, and type IV collagen were significantly higher in db/db mice than in db/m mice (Figure 3). Mesangial expansion was significantly prominent in db/db mice. Further, intrarenal immunostaining for TGF-β, PAI-1, and type IV collagen showed a similar pattern for mesangial expression (Supplementary Figure S2).

### 3.7. VSIG4 Expression in Cultured Podocytes

**Figure 5:**
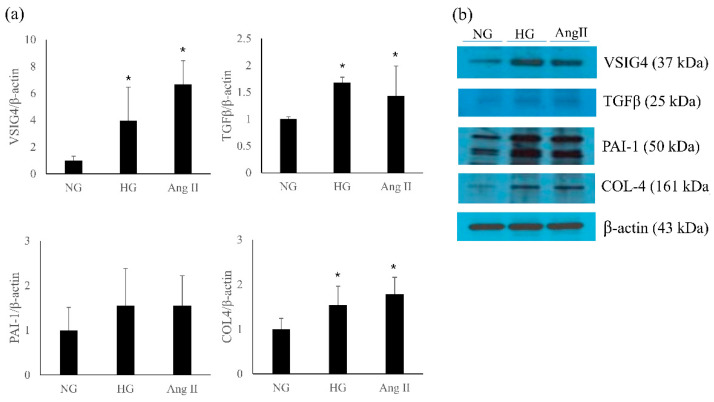
Expression of VSIG4 and profibrotic markers in podocytes stimulated with high glucose (HG) and angiotensin II (Ang II). (**a**) mRNA expression of each marker; (**b**) representative protein expression by Western blot. Values are expressed as mean ± SD. * *p* < 0.05 vs. normal glucose (NG).

The effect of high glucose on VSIG4 and profibrotic markers was determined. In addition, the effect of angiotensin II, a well-known mediator of renal progression, was also examined. Both high glucose and angiotensin II significantly upregulated the expression of VSIG4 mRNA and protein in the podocytes (Figure 5). Further, the role of VSIG4 on the profibrotic markers was determined. The mRNA expression of TGF-β and type IV collagen were significantly upregulated by these treatments. Their protein expression changes were similar to the mRNA expression changes.

## 4. Discussion

The results showed that the mRNA and protein expression of VSIG4 were upregulated in an animal model of type 2 diabetes. Urinary VSIG4 levels were significantly correlated with albuminuria. Furthermore, the upregulated VSIG4 expression was consistent with that of fibrotic markers in this model. In addition, VSIG4 expression was upregulated in podocytes stimulated by high glucose and angiotensin II. To the best of our knowledge, this is the first report of VSIG4 being related to the pathogenesis of DKD in an animal model of type 2 diabetes.

Only a few studies have investigated the role of VSIG4 in DKD. Zeng et al. [16] analyzed the Gene Expression Omnibus database and reported that 15 hub genes were enriched in patients with diabetic nephropathy; VSIG4 was one of the enriched genes associated with the progression of diabetic nephropathy. Xu et al. [17] reported that VSIG4 was upregulated in the glomeruli of diabetic kidneys. Our previous report showed that high glucose stimulated VSIG4 expression in renal tubule cells [12]. In the current study, the expression of VSIG4 and profibrotic markers was upregulated by treatment with high glucose and angiotensin II in cultured podocytes. Combined with the previous results, these findings proved that VSIG4 expression is clearly upregulated in an animal model of type 2 diabetes.

VSIG4 is reported to be associated with EMT in kidney cells and cancer metastasis. Overexpression of VSIG4 induced EMT and significantly promoted invasion and migration of glioblastoma cells [7]. Plasma VSIG4 levels were higher in patients with advanced ovarian cancer and were related to patient survival [18]. Our previous report showed that VSIG4 is involved in Epstein–Barr virus-related EMT in renal tubule cells [11]. Further, VSIG4 was a downstream mediator of TGF-β and an upstream regulator of fibrotic markers in renal tubular cells stimulated by high glucose [12]. Overall, these findings suggest that VSIG4 plays a role in the fibrotic pathway in DKD.

Podocytes are critical cells that maintain glomerular integrity [19]. Podocyte injury contributes to the development and progression of DKD [20]. Our results indicate that high glucose and angiotensin II, a well-known mediator of renal disease progression in diabetes, stimulated VSIG4 and profibrotic marker expression in podocytes. To the best of our knowledge, this is the first report of increased VSIG4 expression in podocytes stimulated by diabetic condition.

In conclusion, VSIG4 was upregulated in the kidneys, as well as in the urine and serum of an animal model of type 2 diabetes. Furthermore, the upregulated expression of VSIG4 was associated with albuminuria and pro-fibrotic markers. Considering these relationships, VSIG4 may be an important mediator in the progression of DKD. Further studies are needed to clarify the relationship of VSIG4 with infiltrated or residual renal macrophages and to clarify the precise mechanisms of VSIG4 in the progression of DKD.

## Figures and Tables

**Table 1:** Biochemical parameters in db/m (n = 6) and db/db (n = 6) mice.

	BW (g)			BS (mg/dL)			HbA1c (%)			Albumin (ug/6 h)		
	8	12	20	8	12	20	8	12	20	8	12	20
db/m	24.9 ± 0.4	27.9 ± 2.7	31.2 ± 2.3	131.5 ± 16.5	149.5 ± 37.6	175.3 ± 13.6	NA	5.23 ± 1.06	4.28 ± 0.18	2.45 ± 1.63	4.93 ± 3.81	3.55 ± 1.47
db/db	34.9 ± 2.0 *	46.1 ± 5.6 *	56.4 ± 5.8 *	480.0 ± 159.6 *	627.7 ± 110.2 *	806.7 ± 286.1 *	NA	10.8 ± 0.84 *	11.0 ± 0.75 *	4.0 ± 4.23	11.36 ± 4.44 *	14.32 ± 2.88 *

Data are presented as the mean ± standard deviation. BW, body weight; BS, blood sugar; NA, not available; * *p* < 0.05.

## Supplementary Materials

The following supporting information can be downloaded at: https://www.mdpi.com/article/10.3390/life12071031/s1, Figure S1: Representative immunohistochemical staining for VSIG4; Figure S2: Representative immunohistochemical staining. (A) PAS; (B) TGF-β: (C) PAI-1: (D) type IV collagen. Original magnification ×400. (E) Semiquantitative expression of each molecule * *p* < 0.05 vs. db/m; Table S1: Primer sequences for quantitative RT-PCR.

## Abbreviations

VSIG4	V-set Ig domain containing 4
DKD	diabetic kidney disease
EMT	epithelial-mesenchymal transition
TGF-β	transforming growth factor-β
PAI-1	plasminogen activator inhibitor-1
