# Supplementary material for: Upregulation of VSIG4 in Type 2 Diabetic Kidney Disease
Source: Life (Basel). 2022 Jul 11;12(7):1031. doi: 10.3390/life12071031 (PMC9318196; doi:10.3390/life12071031)

Supplementary Table S1. Primer sequences for quantitative RT-PCR.

Target gene	Primer sequence (5’ to 3’)
VSIG4, forward	TCCCTGGCTTCCTTTCTTCT
VSIG4, reverse	CCA AAC CCA GGA TTT CTC AA
TGFβ, forward	AGCCCGAAGCGGACTACTAT
TGFβ, reverse	CTGTGTGAGATGTCTTTGGTTTTC
PAI-1, forward	TCCTCATCCTGCCTAAGTTCTC
PAI-1, reverse	GTGCCGCTCTCGTTTACCTC
Col 4, forward	GCTCTGGCTGTGGAAAATGT
Col 4, reverse	CTTGCATCCCGGGAAATC
β-actin, forward	GGACTCCTATGTGGGTGACG
β-actin, reverse	CTTCTCCATGTCGTCCCAGT

Supplementary Figure S1. Representative immunohistochemical staining for VSIG4.

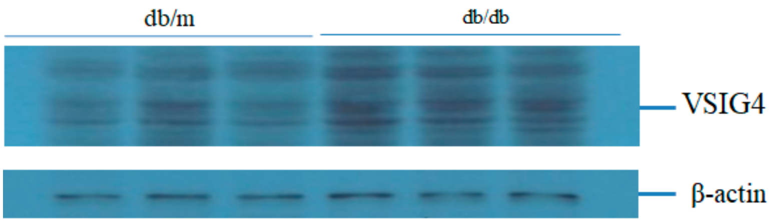

Supplementary Figure S2. Representative immunohistochemical staining. (A) PAS; (B) TGF-β: (C) PAI-1: (D) type IV collagen. Original magnification x 400. (E) Semiquantitative expression of each molecule * $P < 0.05$ vs. db/m.

(db/m) (db/db)

(A) (B) (C) (D)

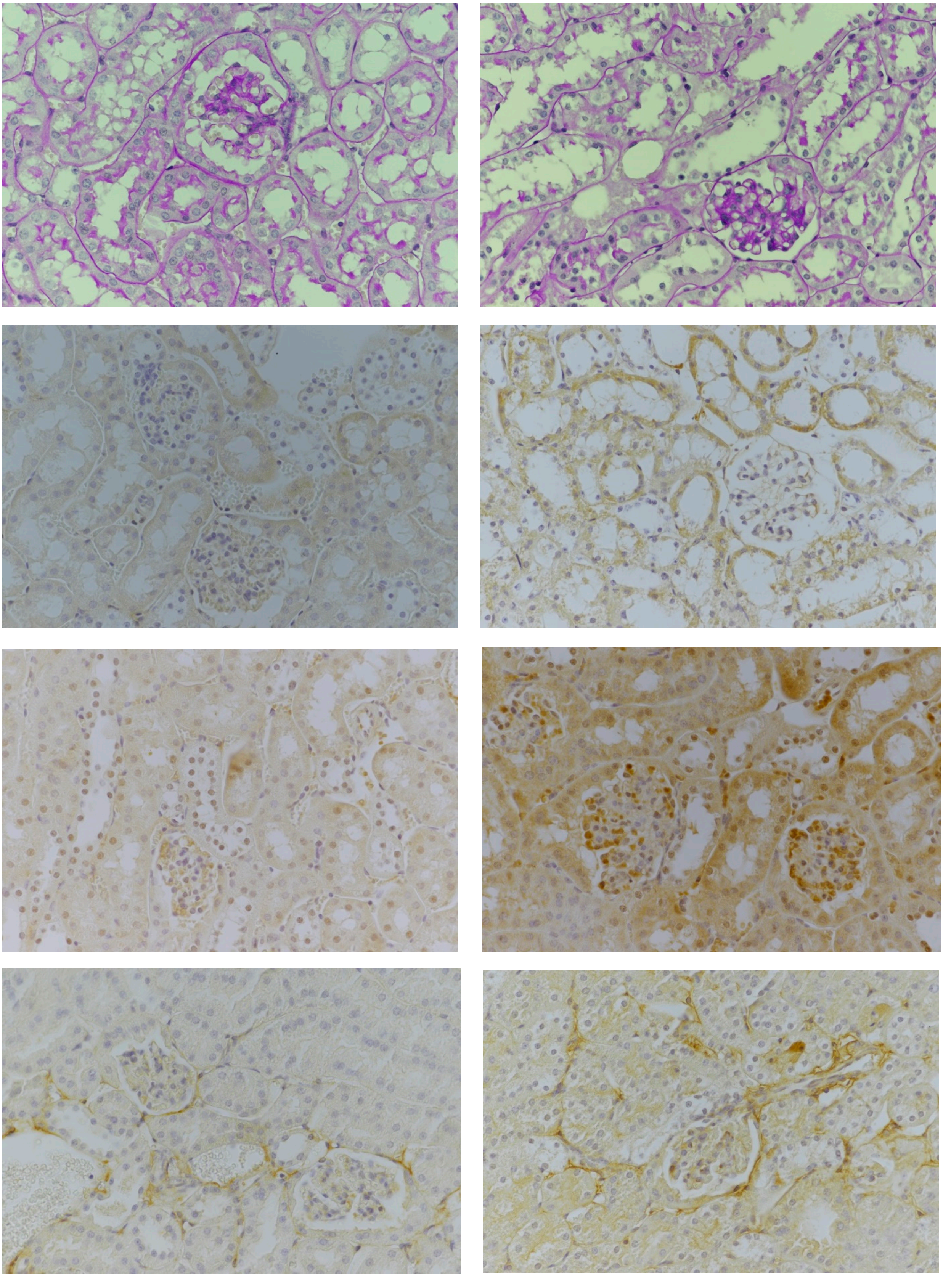

(E)

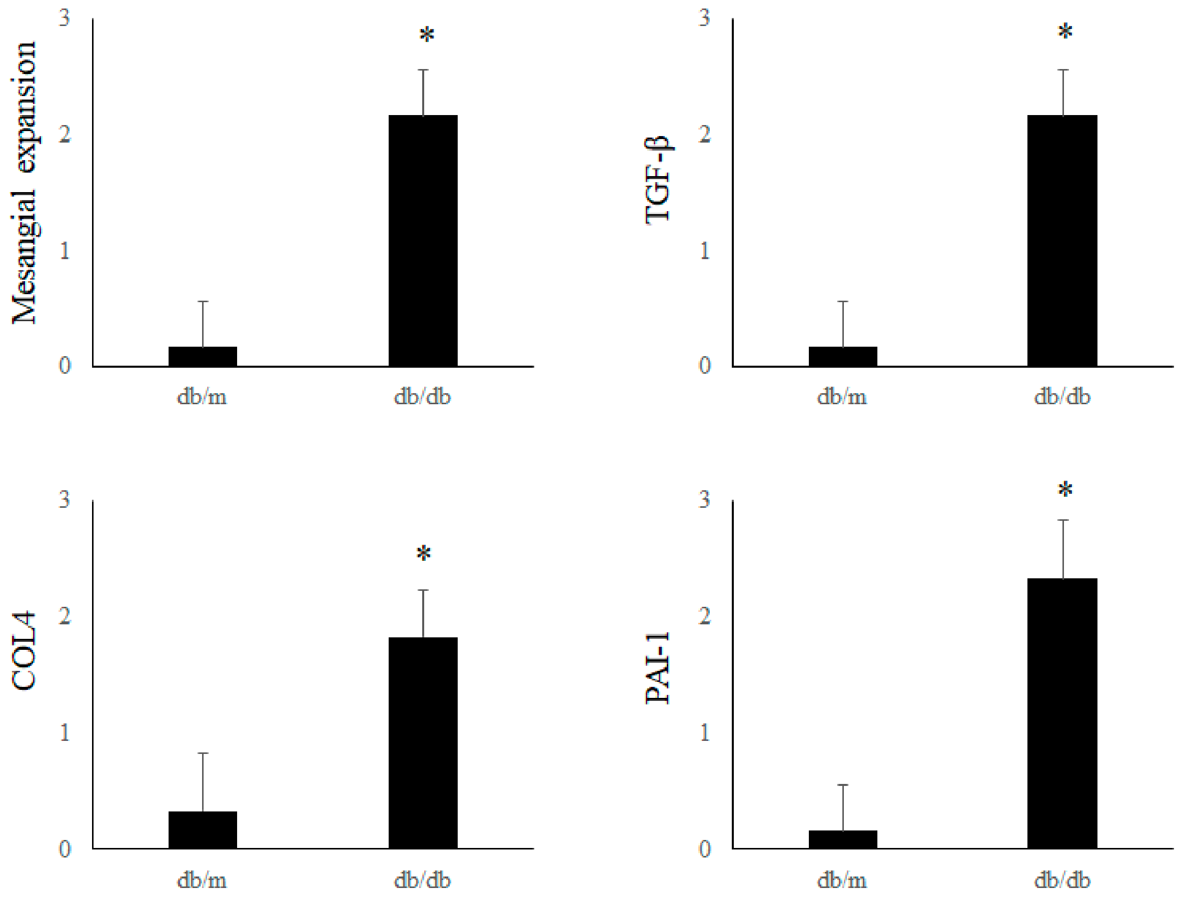

Supplement: Supplementary file 1 [file life-12-01031-s001.zip › life-1797537-supplementary.pdf]
